# Mechanism and Component Study of Scorpion Peptides Regulation of Macrophage Polarization in Diabetic Wounds

**DOI:** 10.3390/biomedicines14092071

**Published:** 2026-09-15

**Authors:** Si Shi, Yarong Ding, Shuangxi Yang, Furong Zhu, Li Chen, Xinling Huang, Zhongzhi Zhou

**Affiliations:** 1 Department of Burn Plastic Surgery, The First Hospital of Hunan University of Chinese Medicine, Changsha 410007, China; 13137916197@163.com (S.S.); dingyarong@hnucm.edu.cn (Y.D.); 18274864424@163.com (S.Y.); zyfyzczbb@163.com (F.Z.); 2 Medical College, Hunan University of Chinese Medicine, Changsha 410208, China

**Keywords:** scorpion peptides, diabetic wound healing, peptideomics, macrophage polarization, inflammatory response

## Abstract

**Background:** The incidence of diabetes-related chronic wounds has been increasing annually, characterized by delayed wound healing due to persistent inflammatory responses. To alleviate patient suffering, there is an urgent need to discover novel wound-healing agents that reduce local inflammatory reactions. Animal peptides emerge as promising candidates due to their low molecular weight, low toxicity, low resistance potential, high sensitivity, potent activity, and ease of transmembrane absorption. However, the precise mechanism by which animal peptides accelerate skin wound healing remains unclear. **Method:** Scorpion peptides were extracted using ultrafiltration, followed by de novo analysis after library searching with Peaks 8 software and combined LC-MS/MS detection to identify peptide components in scorpions. A full-thickness skin defect model in diabetic mice was established to evaluate the wound-healing promotion capacity of scorpion peptides. In vitro experiments were conducted using mouse monocyte–macrophage leukemia cells (RAW264.7 cells). Enzyme-linked immunosorbent assay (ELISA) evaluated inflammatory cytokine expression, flow cytometry analyzed macrophage phenotypes, Western blotting (WB) measured P-P65 and P65 protein levels, immunofluorescence to observe P-P65 nuclear translocation, molecular docking and molecular dynamics simulations, and the Cellular Thermal Shift Assay (CETSA) to evaluate the binding capacity of scorpion peptides to target proteins, and the TUNEL assay to detect apoptosis, among other methods, to investigate the mechanisms by which scorpion peptides promote wound healing. **Result:** This study successfully identified 2331 peptides from a scorpion peptide extract. In a full-thickness skin defect model in diabetic mice, treatment with scorpion peptides significantly promoted wound healing and induced M2 polarization of macrophages at the wound site. Further in vitro mechanism studies were conducted using a RAW264.7 cell inflammation model established by combining lipopolysaccharide (LPS) with high glucose. The results showed that scorpion peptide reduced the pro-inflammatory cytokine tumor necrosis factor-α (TNF-α), increased the anti-inflammatory cytokine interleukin-10 (IL-10), downregulate the P-P65/P65 protein expression ratio, and reduce the number of TUNEL-positive apoptotic cells, suggesting that the NF-κB pathway may be involved in mediating its anti-inflammatory and anti-apoptotic effects. To further identify the target, molecular docking and molecular dynamics simulations showed that the active peptide segments PPPPPP and GPPPPP adopt stable binding conformations with the P65 protein; CETSA further validated that they enhance the thermal stability of the P-P65 protein. Through repeated validation using active peptide fragments, ELISA, flow cytometry, Western blot, and immunofluorescence assays consistently confirmed that scorpion peptide, PPPPPPP, and GPPPPP not only inhibit P65 phosphorylation but also inhibit the nuclear translocation of P-P65, and exhibit anti-inflammatory, macrophage M2 polarization-promoting, and anti-apoptotic effects. **Conclusions:** Scorpion peptides and their active peptide fragments block the activation of the NF-κB pathway by interfering with P65 phosphorylation and nuclear translocation, effectively inducing M2 polarization of macrophages, reducing inflammation and apoptosis, and ultimately promoting the healing of diabetic wounds.

## 1. Introduction

Diabetic ulcers (DUs) represent one of the primary complications arising in the terminal stages of diabetes, caused by prolonged exposure to a highly pathological hyperglycemic environment. Epidemiological statistics indicate that approximately 537 million individuals worldwide have diabetes, with 19–34% of these patients developing diabetic ulcers [1,2]. Diabetic ulcers are characterized by complex etiology, high incidence, recurrent nature, chronicity, and treatment challenges. They frequently progress to chronic non-healing wounds, ultimately leading to local necrosis and amputation, imposing dual psychological and physiological burdens on patients [3]. Wound repair progresses through three stages: inflammation, proliferation, and remodeling [4]. Research indicates that delayed inflammatory responses and dysregulation of inflammatory factors are key factors contributing to the poor healing of diabetic wounds [5,6]. Macrophages, as vital immune cells, play crucial roles in clearing infectious agents, mediating inflammatory responses, and regulating remodeling and repair [7]. Their regulatory functions span the entire wound healing process, playing a primary role in chronic wound progression and serving as key cells in wound repair [8]. M1-type macrophages produce high levels of inducible nitric oxide synthase (iNOS), highly express various receptors such as CD80 and CD86, and secrete large amounts of pro-inflammatory factors including TNF-α, IL-6, and NO. M2 macrophages highly express arginase (primarily Arg-1) and synthesize polyamines and ornithine via the arginine pathway, providing raw materials for subsequent cell proliferation and collagen synthesis. They also highly express various receptors, such as CD206 and CD163, and produce anti-inflammatory factors such as IL-10 and TGF-β1 [9].

During the normal healing process, the wound transitions from the inflammatory phase to the proliferative phase, and macrophages in the wound tissue switch from the pro-inflammatory M1 phenotype to the anti-inflammatory M2 phenotype, producing mediators and growth factors that promote repair. Vascular endothelial growth factor (VEGF) promotes angiogenesis and tissue oxygenation between days 3 and 7. Proliferating cell nuclear antigen (PCNA) promotes re-epithelialization and active granulation tissue proliferation on day 3 [10]. Type III collagen forms a temporary scaffold in the early stages of wound healing and is gradually replaced by type I collagen during the remodeling phase, leading to the reconstruction of the extracellular matrix (ECM) and enhancing tensile strength [11].In diabetic conditions, the wound microenvironment is abnormal, and the phenotypic conversion of macrophages is strongly inhibited, causing them to remain in the pro-inflammatory M1 phenotype for prolonged periods. At this stage, M1 macrophages exhibit greater pro-inflammatory activity; massive infiltration of inflammatory cells leads to an excessive inflammatory response, preventing an orderly transition from the inflammatory phase to the proliferative phase. Reduced expression of VEGF and PCNA, along with decreased collagen and an imbalanced I/III ratio, hinder effective wound healing [12].

Current primary external treatments for diabetic wounds in clinical practice include medication, topical dressings, surgical debridement, and negative pressure wound therapy. Despite the variety of approaches, challenges persist, such as suboptimal therapeutic outcomes and high costs [13]. Peptides offer therapeutic advantages such as low molecular weight, low toxicity, high activity, and high affinity, enabling them to achieve higher concentrations in superficial tissues [14]. This makes them suitable for preparing topical formulations for skin wounds, allowing for localized delivery through the biological barrier [15]. JDSJG (Jie Du Sheng Ji Gao; First Affiliated Hospital of Hunan University of Chinese Medicine, China) is a proprietary Chinese herbal ointment. Previous studies demonstrate its superior therapeutic efficacy for diabetic skin wounds by downregulating inflammatory factors such as IL-6 and TNF-α, promoting granulation tissue growth in diabetic rat wounds, and accelerating wound healing [16,17,18]. Scorpion peptides, the primary medicinal active components of scorpions, have been demonstrated as a rich source of bioactive peptides with therapeutic potential, exhibiting significant anti-inflammatory, antimicrobial, and immunomodulatory effects [19,20,21]. Studies by Arianna et al. showed that scorpion venom at varying concentrations and incubation times reduced cytokine levels of IL-6, IL-1β, and IFN-γ [22]. Xu E et al. confirmed that scorpion extracts reduce macrophage inflammation by targeting Kv1.3 channels [23]; Jantaruk P et al. found that antimicrobial peptides extracted from scorpions significantly inhibit the production of inflammatory mediators nitric oxide and IL-6 in macrophages induced by Pseudomonas aeruginosa LPS [24]. Current research on scorpions primarily focuses on their roles in antitumor effects, ion channel regulation, and stimulation of nerve conduction [20,25]. The specific mechanisms by which scorpions and their active components modulate macrophage polarization and alleviate persistent inflammatory responses in diabetic wounds remain unclear and warrant further investigation.

This study provides conclusive evidence of scorpion peptide’s exceptional wound-healing properties, demonstrating its active role in regulating inflammatory factors and macrophage polarization to promote wound healing. It presents a high-quality, promising candidate for enhancing wound healing in diabetic conditions.

## 2. Materials and Methods

### 2.1. Preparation of Scorpion Peptides and Determination of Their Concentration and Heavy Metal Content

Scorpions were purchased from the First Affiliated Hospital of Hunan University of Chinese Medicine (Changsha, China). The whole scorpion peptides and single peptide components were extracted by GenScript Biotech Corporation (Nanjing, China). Tail and abdominal tissues were excised from the scorpion and crushed into a granular powder. The powder was soaked in >95% ethanol and sonicated for 2 h, repeated 3–4 times. After preliminary peptide content assessment via liquid chromatography, the extract was filtered to remove solid residues and evaporated under reduced pressure at 35 °C until nearly dry. A small amount of extract was dissolved in 99.9% pure ethanol, refrigerated, and allowed to stand for 2–3 days to precipitate white solid peptides. Discard the supernatant containing lipid-soluble impurities, resuspend the precipitate in an appropriate amount of pure water, and use this as the feed solution for membrane separation. This resuspension is sequentially subjected to tandem fractionation using a modified polyethersulfone ultrafiltration membrane with a molecular weight cut-off of 30 kDa (first step: coarse filtration to remove residual high-molecular-weight impurity proteins and insoluble fibrous materials) and a regenerated cellulose ultrafiltration membrane with a molecular weight cut-off of 3 kDa (second step: fine fractionation, which retains and enriches the target component while removing small-molecule impurities such as free amino acids and inorganic salts) in a series configuration. The permeate with a molecular weight of 3 kDa or less is collected, freeze-dried to obtain scorpion peptide lyophilized powder, and the stability of the extract is verified through liquid-phase analysis. The concentration of the scorpion peptide fragments was determined using the BCA method, and a portion of the sample was sent to Hangzhou Yanqu Information Technology Co., Ltd. (Hangzhou, China) for heavy metal content analysis.

### 2.2. LC-MS/MS Analysis

Add an appropriate amount of 0.1% formic acid to dissolve the peptide segments. Desalt the sample using a C18 desalting column. Activate the desalting column with 100% acetonitrile, equilibrate the column with 0.1% formic acid, load the sample onto the column, then wash the column with 0.1% formic acid to remove impurities. Finally, elute with 70% acetonitrile, collect the flow-through, and freeze-dry. Prepare mobile phase A (100% water, 0.1% formic acid) and B (80% acetonitrile, 0.1% formic acid). Dissolve lyophilized powder in 10 µL of A, centrifuge at 14,000 g for 20 min at 4 °C, and inject 1 µg of supernatant for LC-MS analysis. Employ the Q Exactive HF-X mass spectrometer (Thermo Fisher Scientific, MA, USA) with a Nanospray Flex™ (NSI) ion source. Set ion spray voltage to 2.4 kV and ion transfer tube temperature to 275 °C. Utilize data-dependent acquisition mode with a full scan range of *m*/*z* 350–1500. with primary mass resolution set to 120,000 (at 200 *m*/*z*), AGC at 3 × 10^6^, and maximum C-trap injection time of 80 ms. The top 40 parent ions with the highest intensity in the full scan were selected for fragmentation using high-energy collision-induced dissociation (HCD) for secondary mass spectrometry detection. The secondary mass spectrometry resolution was set to 15,000 (at 200 *m*/*z*), with an AGC of 5 × 10^4^, a maximum injection time of 45 ms, and a peptide fragmentation collision energy of 27%. Raw mass spectrometry detection data (.raw) were generated.

### 2.3. Database Search and De Novo Protein Sequencing

When searching the database using Peaks 8 software, follow these principles: For sequenced organisms, directly select the species-specific database; for non-sequenced organisms, choose the broadest proteome database most relevant to the tested sample. Perform de novo protein sequencing analysis on the identified peptides.

### 2.4. Preparation of Hydrogels

Pluronic^®^ F-127 (P2443, Sigma-Aldrich, MA, USA) is a polyethylene oxide-polypropylene oxide-polyethylene oxide (PEO-PPO-PEO) blend containing hydrophobic (PPO) and hydrophilic (PEO) segments [26]. F127 is thermosensitive; it remains in the liquid phase at low temperatures (≤10 °C), transforms into a gel at temperatures above 20 °C, and forms a stable, porous, three-dimensional hydrogel network at 37 °C [27]. F127 hydrogel biomaterials have garnered significant attention in wound healing due to their remarkable properties, including temperature sensitivity, biodegradability, and the ability to maintain a moist wound environment [28,29]. Prepare a 5% (*w*/*v*) hydrogel by mixing F127 with deionized water. Place the mixture in a 4 °C refrigerator and let it stand for several hours; gentle agitation may be used to accelerate dissolution until a clear, particle-free, transparent liquid forms.

### 2.5. Laboratory Animals

SPF-grade male C57BL/6J mice (20–30 g, 6–8 weeks old) were purchased from Tianqin Biotechnology Co., Ltd. (Changsha, China). Housing conditions: temperature 20–22 °C, relative humidity 40–60%, 12 h light/12 h dark cycle. All mice underwent a one-week acclimatization period prior to experimentation. All animal experiments were conducted at the Animal Experimentation Center of the First Affiliated Hospital of Hunan University of Chinese Medicine. The experimental protocol was reviewed and approved by the Animal Experimentation Ethics Committee of the First Affiliated Hospital of Hunan University of Chinese Medicine, under approval number (202503357). The development of animal models and the conduct of the experiments strictly adhered to ethical guidelines for animal research.

### 2.6. Diabetic Mouse Full-Thickness Skin Defect Model

Diabetes modeling group: After 14 days of high-fat diet feeding, mice received intraperitoneal injections of streptozotocin (STZ; Solarbio, Beijing, China) at 180 mg/kg for 3 consecutive days. Random blood glucose levels were measured via tail vein blood sampling. Mice with blood glucose levels ≥ 16.7 mmol/L for 3 consecutive days were considered successfully modeled for diabetes [30,31]. After the model was successfully established, the diabetic mice gradually developed the classic “three excesses and one deficiency” symptoms (excessive thirst, excessive appetite, excessive urination, and weight loss). Changes in body weight and blood glucose levels are shown in Supplementary Figure S1.

After intraperitoneal anesthesia with 1% sodium pentobarbital, the dorsal neck region was shaved to create a 3 cm × 3 cm square denuded skin area. The site was sequentially disinfected with povidone-iodine and 75% ethanol. Using a punch biotome from Haopai Biotechnology Co., Ltd. (Zhengzhou, China), two 1 cm diameter full-thickness circular skin defects were created along the dorsal spine at the cervical-dorsal region. The model was considered successful if the wound exhibited diffuse, flattening, slow granulation tissue growth, and pallor, the full-thickness skin defect model in diabetic mice was considered successful.

### 2.7. Animal Experiment Grouping and Treatment

Twenty-four SPF-grade C57BL/6J mice were randomly assigned using a random number table to a normal group (*n* = 6) and a diabetes model group (*n* = 18). Mice successfully modeled for diabetes were randomly divided into a model group, a JDSJG group, and a scorpion peptide group, with six mice in each group. Following routine disinfection, the control group and model group wounds were applied with 200 μL of blank hydrogel. The JDSJG group wounds were locally covered with approximately 2 mm thick JDSJG medication, fully covering the wound surface. The scorpion peptide group wounds were applied with 200 μL of 0.5 mg/mL scorpion peptides hydrogel. Wound dressings were changed once daily for 14 consecutive days. Photographs of the wounds were taken at 0, 3, 7, and 14 days post-dressing change to observe wound healing in each group. Independent researchers, who were unaware of the group assignments, took photographs of the wounds and used the image analysis software ImageJ version 1.54t (Bethesda, MD, USA) to measure the wound area and calculate the wound healing rate. Wound healing rate = [(Original wound area − Wound area at corresponding observation time)/Original wound area] × 100%.

In this study, individual mice were treated as independent statistical units (*n* = 6 per group). On day 14, tissue samples were collected from the wound site; mice were anesthetized by intraperitoneal injection of 1% sodium pentobarbital and then euthanized by decapitation. Full-thickness skin tissue specimens measuring approximately 1 cm × 1 cm were collected from the center of the wound site. One specimen from each side was fixed in a bottle of 4% paraformaldehyde and stored in a 4 °C refrigerator, while the other specimen from each side was cryopreserved in a −80 °C freezer.

### 2.8. Molecular Docking

Using the AlphaFold3 tool, binding mode predictions were performed for the full-length P65 sequence and its top-performing active peptides [32,33,34]. The protein amino acid sequence of the target protein P65 was obtained from the UniPort protein database (UniPort, https://www.uniprot.org/). AlphaFold3′s deep learning model was employed for three-dimensional structure prediction. Following energy optimization and molecular dynamics simulations, the optimal protein conformation was determined and active sites were extracted. Subsequently, PeptideBuilder was used to construct the three-dimensional structure of the target peptide and perform energy minimization. Finally, the preprocessed P65 protein and peptides were imported into the docking module. Rigid docking calculations were performed with binding free energy as the core evaluation metric to screen for docking complexes with optimal binding conformations, providing a structural foundation for subsequent interaction mechanism analysis.

### 2.9. Molecular Dynamics

Molecular dynamics simulations were performed using GROMACS software version 2026.3 (Stockholm, Sweden). Both the P65 protein and the peptide segment composed of standard amino acids were parameterized using the AMBER99SB-ILDN force field. The system was solvated using the TIP3P explicit water model, and an appropriate number of counterions were added under periodic boundary conditions to achieve electrical neutrality. First, an energy minimization was performed to eliminate unfavorable atomic contacts. Subsequently, the system was equilibrated to 300 K under the NVT ensemble and then maintained at 300 K and 1 bar under the NPT ensemble to achieve density equilibrium. Based on this, a 100 ns productive molecular dynamics simulation was conducted with an integration time step of 2 fs. Long-range electrostatic interactions were handled using the Particle Mesh Ewald (PME) method, with the cutoff distances for electrostatic real-space and van der Waals interactions set to 1.0 nm according to the simulation parameters. After correcting the trajectories for periodic boundary conditions and fitting them to the protein structure, the RMSD and RMSF of the protein and peptide segments, as well as the number and occupancy of protein-peptide hydrogen bonds, were calculated to evaluate the conformational changes of the complex and the persistence of interfacial interactions. Concurrently, the MM/GBSA method was used to compare trends in relative binding energies. The P65-active peptide system was subjected to a consistent simulation and analysis workflow for cross-comparison, in which only inactive peptides confirmed in advance by experiments or research design were defined as negative controls.

### 2.10. CETSA

After treating the cells, wash them with phosphate-buffered saline (PBS) and centrifuge to collect the cell pellet; then, resuspend the pellet in a PBS solution containing a mixture of protease and phosphatase inhibitors. The protein samples were heated for 3 min at a predetermined temperature gradient (37 °C, 42 °C, 47 °C, 52 °C, 57 °C, and 62 °C). After the samples cooled to room temperature, they were immediately flash-frozen in liquid nitrogen, then thawed in a water bath; this freeze–thaw cycle was repeated three times. Centrifuge the samples for 30 min at 4 °C and 12,000 rpm to collect the supernatant. Perform Western blotting analysis on the protein supernatant and use ImageJ software to quantitatively analyze the band intensities.

### 2.11. Cell Culture

RAW264.7 cells were purchased from Starfish Biotech (TCM-C766-UD, Suzhou, China). RAW264.7 cells were thawed and cultured in DMEM medium (DMEM; Gibco, Suzhou, China) supplemented with 10% fetal bovine serum (FBS; Hyclone, Logan, UT, USA). Cells were cultured in a 37 °C incubator with 5% CO_2_, 95% air, and saturated humidity. Experiments were conducted when cells reached 80–90% confluence. The control group was cultured in DMEM containing 10% FBS, while other groups received specific interventions as required.

### 2.12. Cell Experiment Grouping and Intervention

The study groups were divided into a control group, a model group, a scorpion peptide group, and an active peptide fragment group. Except for the control group, all other groups underwent modeling intervention with 1 μg/mL LPS (Sigma-Aldrich, Shanghai, China) plus 30 μmol/mL high glucose. The drug concentrations and intervention durations for scorpion peptides and active peptide fragments were determined based on preliminary CCK8 assays.

### 2.13. CCK8

Seed 1.5 × 10^5^ RAW264.7 cells into a 96-well plate and incubate for 24 h. Set up a normal group, model group, and scorpion peptides concentration groups (0.05, 0.1, 0.5, 1, 2.5, 5, 10 μg/mL), as well as active peptide segment concentration groups (0.05, 0.1, 0.5, 1, 5, 10, 20 μg/mL), and incubate for 24 h. Add Cell Counting Kit-8 (CCK-8; Beyotime, Shanghai, China) and incubate for 30 min. Measure absorbance at 450 nm wavelength to assess the effects of different scorpion peptides and active peptide concentrations on macrophage proliferation activity. Cell survival rate = [(Experimental group OD value − Blank group OD value)/(Control group OD value − Blank group OD value)] × 100%.

### 2.14. Enzyme-Linked Immunosorbent Assay (ELISA)

Cell supernatants were centrifuged at 1000 rpm for 5 min at 4 °C. The supernatants were collected and used to detect the expression levels of TNF-α and IL-10 in the samples according to the instructions of the ELISA kit (MULTI SCIENCES, Hangzhou, China). The quantitative levels of inflammatory cytokines were calculated based on the standard curve. Set up standard and sample wells. Add 50 μL and 100 μL of the standard solution at different concentrations to the standard wells and add 100 μL of the enzyme-linked reagent to these wells. Set up blank wells and wells for the samples to be tested. To the sample wells, add 40 μL of diluent, 10 μL of the cell supernatant to be tested, and 100 μL of ELISA reagent, in that order. Seal the microplate with a film and incubate in a 37 °C incubator for 60 min. Discard the liquid, fill each well to the brim with wash buffer, let stand for 30 s, then discard. Repeat this washing step five times. Add the color developer and mix thoroughly; allow the reaction to proceed in the dark at 37 °C for 15 min. Finally, add 50 μL of stop solution, zero the reading using the blank well, and measure the OD values of each well at 450 nm using a microplate reader. Calculate the concentration of the target factor in the cell supernatant based on the standard curve.

### 2.15. Western Blotting (WB)

Collect cells, lyse them with PIRA lysis buffer, extract total cellular protein, and determine protein concentration using the BCA method. Separate protein samples by SDS-PAGE electrophoresis, then transfer them to a PVDF membrane. Block the membrane at room temperature for 1 h using a buffer containing skim milk powder. Primary antibodies (NF-κB P65 antibody, Proteintech, 80979-1-RR, 1:40,000, IL, USA; NF-κB P-P65 antibody, Abcam, ab76302, 1:1000, Cambridge, UK) were added and incubated overnight at 4 °C. The primary antibody incubation solution was discarded, and secondary antibodies (HRP-labeled Goat Anti-Rabbit IgG, A21020, Abbkine, Wuhan, China; HRP, Goat Anti-Mouse IgG, A21010, Abbkine, Wuhan, China) and incubate at room temperature for 2 h. Detect using a chemiluminescence system (Image Lab, Bio-Rad, Hercules, CA, USA). Repeat the experiment three times to detect the expression levels of P65 and P-P65 proteins.

### 2.16. Immunofluorescence

Seed the cells in confocal culture dishes and culture them overnight to allow the cells to fully adhere to the surface. After treating the cells with the appropriate drug for 24 h, discard the culture medium and wash the cells three times with pre-chilled PBS. Fix the cells with 4% paraformaldehyde at room temperature for 15 min. After washing with PBS, perform a permeabilization step with PBS containing 0.1–0.3% Triton × -100 for 10 min. Subsequently, the cells were blocked with a blocking solution containing 5% BSA at room temperature for 1 h. After blocking, the anti-phosphorylated NF-κB P65 (Ser468) primary antibody (Phospho-NF-κB P65 (Ser468) recombinant monoclonal antibody, AB_3083091, Proteintech, Rosemont, IL, USA) was added, and the sample was incubated overnight at 4 °C. The next day, after thorough washing with PBS, the fluorescently labeled secondary antibody (rabbit IgG (H + L), DyLight 488, A23220, Abbkine, Wuhan, China) was added in the dark and incubated at room temperature for 1 h. After washing with PBS, the cell nuclei were counterstained with DAPI (0.2 ng/mL) for 5–10 min. After mounting the slides, images were acquired using a ZEISS (LSM800, TH, Carl Zeiss Microscopy GmbH, Jena, Germany) confocal microscope.

### 2.17. Flow Cytometry

For wound tissue, the collected mouse wound tissue was trimmed and rinsed twice in pre-chilled PBS to remove red blood cells and impurities. The tissue was then cut into small fragments of approximately 1 mm^3^ and digested for 60 min at a constant temperature of 37 °C with agitation in a digestive solution containing a final concentration of 0.3 mg/mL collagenase I and collagenase IV (Sigma-Aldrich, shanghai, China) at a final concentration of 0.3 mg/mL. After digestion was terminated, 10 mL of pre-chilled PBS was added, and the mixture was transferred to a 15 mL centrifuge tube. The tube was centrifuged at 350 g for 10 min; the supernatant was removed, leaving 300 μL. The remaining volume was mixed, filtered through a 70 μm cell strainer, and the filtrate was centrifuged to obtain a tissue-derived single-cell suspension. For adherent macrophages induced to differentiate in vitro, discard the culture supernatant and gently wash twice with pre-chilled PBS. Use a sterile cell scraper to detach the adherent cells, collect the cell suspension, centrifuge and wash it, then resuspend for later use.

Add the following fluorescently labeled antibodies to the collected cell suspensions for surface staining: First, add the LIVE/DEAD^TM^ Fixable Aqua Live/Dead Staining Kit (Invitrogen, catalog number L34966, Frederick, MD, USA) to label live and dead cells and incubate for 15 min. Next, add the FITC-labeled anti-mouse CD11b antibody (BD Biosciences, catalog number 557396, San Jose, CA, USA), the PE-labeled anti-mouse CD86 antibody (BD Biosciences, catalog number 561963), the PE-Cyanine 7-labeled anti-mouse F4/80 antibody (BioLegend, catalog number 123113, San Diego, CA, USA), PE-labeled anti-mouse CD206 antibody (BD Biosciences, catalog number 568808), and APC-Cy7-labeled anti-mouse CD45 antibody (BD Biosciences, catalog number 561037). Incubate the antibodies at 4 °C in the dark for 30 min. After staining, wash and fix the samples. Data were collected using a flow cytometer (BD Canto II, San Jose, CA, USA), and flow cytometry data were analyzed using FlowJo software (version 10.8.1). See Supplementary Figure S2 and Table S1 for specific gating strategies and representative flow cytometry plots.

### 2.18. TUNEL

TUNEL assay (MeilunBio, MA0224, Dalian, China) was used to detect DNA fragmentation. Macrophages were seeded into 6-well plates and cultured for 24 h. Subsequently, cells were treated with drugs for 24 h according to different groups. Cells were fixed with 4% paraformaldehyde and incubated according to the manufacturer’s instructions, then observed using a ZEISS confocal microscope.

### 2.19. Statistical Analysis

Data are expressed as mean ± standard deviation (SD). Normality was assessed by Shapiro–Wilk test. For single-time-point comparisons, two-group data were analyzed by independent *t*-test, and multi-group data by one-way ANOVA with Tukey′s post hoc test. For longitudinal wound area data, a linear mixed-effects model was used, with treatment and time as fixed effects and individual mouse as random effect, followed by Bonferroni-corrected pairwise comparisons. All analyses were performed using SPSS (version 26.0), and *p* < 0.05 was considered significant.

## 3. Results

### 3.1. Scorpion Peptides Extraction and Identification

**Figure 1:**
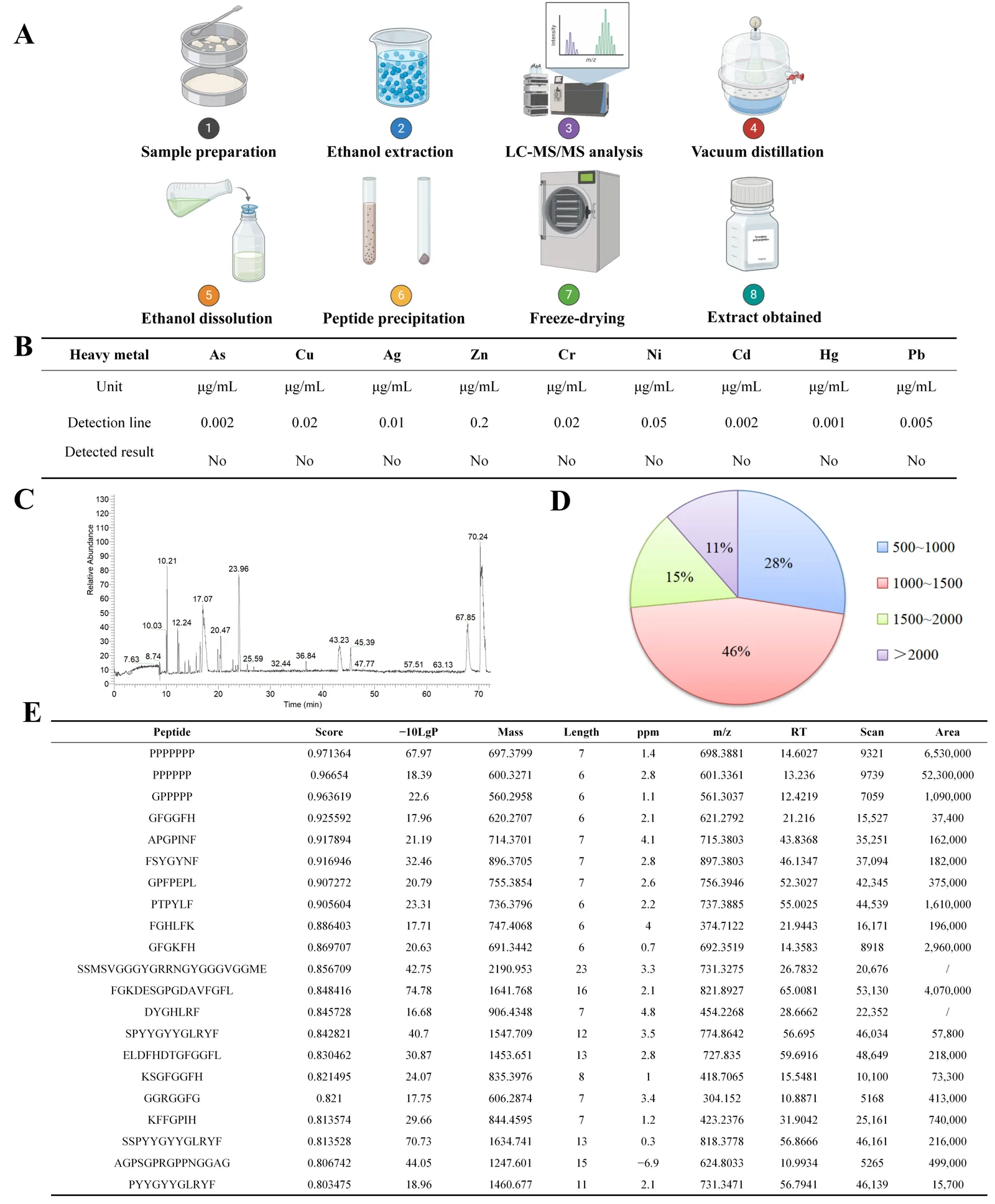
(**A**) Flowchart of scorpion peptides extraction. (**B**) Determination results of mineral and heavy metal content. (**C**) Total ion chromatogram of scorpion peptides. (**D**) Molecular weight distribution of scorpion peptides. (**E**) Basic characteristics of peptide segments with PR values ≥ 0.80, including peptide activity scores, molecular weight, sequence length, etc. Here, “Peptide” denotes the peptide sequence, “−10lgP” represents the significance score of peptides identified via database searches or de novo sequencing, ppm denotes the error between detected and theoretical molecular weight, *m*/*z* indicates the peptide mass-to-charge ratio, RT represents retention time, Scan corresponds to the spectrum number, and Area signifies peak area (typically the peak area of the peptide associated with the optimal PSM).

Scorpion peptides were obtained via ultrafiltration as peptides with molecular weights below 3500. The concentration of the scorpion peptide segment was determined to be 1 mg/mL using the BCA method (Figure 1A). Under optimized process conditions (0.3 M NaOH for alkali treatment, 4 h of enzymatic hydrolysis, 0.3 MPa membrane separation pressure, and a sealed environment maintained at a constant temperature of 10 °C throughout the process), the total recovery rate of this combined process reached 91.2%, representing an increase of approximately 35% compared to the traditional solvent extraction method; small-molecule oligopeptides accounted for ≥80% of the final product, with protein purity and the purity of the target active peptides both ≥90%; the molecular weight was concentrated in the 500–1000 Da range, consistent with the absorption characteristics of small-molecule active peptides. Results from six consecutive scale-up validation batches showed that the RSD for peptide yield was 2.17% and the RSD for purity was 1.89%. Inter-batch variations in product appearance, solubility, and activity parameters were all controlled within 5%, confirming that the integration of ethanol precipitation and ultrafiltration was seamless. The overall process demonstrated stability and good reproducibility, meeting the quality control requirements for subsequent pilot-scale upscaling. Heavy metal content detection results indicated that the heavy metal levels in the scorpion peptides were all within the machine detection error range, thus the impact of heavy metals on cells could be considered negligible (Figure 1B). Preliminary identification of scorpion peptides was performed using LC-MS/MS, yielding a total ion current (TIC) profile (Figure 1C). Peak identification was performed using Peaks 8 software for database searching and de novo protein sequencing. A total of 5537 primary mass spectra and 56,644 secondary mass spectra were identified. The de novo identified peptides numbered 2331, while database-matched peptides totaled 1583. Evaluation of the molecular weight distribution of peptides revealed a range of 515–3450 Da, with a peak concentration between 1000–1500 Da. Peptides within the 500–1500 Da range constituted 73.5% of the total (Figure 1D). with sequence lengths ranging from 7 to 29 amino acids. Bioinformatics tools such as PeptideRanker have become powerful methods for predicting bioactive peptides from proteins [35,36]. The PeptideRanker web server (http://distilldeep.ucd.ie/peptideranker, accessed on 3 December 2025) was used to predict the potential biological activity of the peptides, selecting those with high Peptide Ranker (PR) scores. When PR ≥ 0.50, the peptide was labeled as bioactive; higher PR values indicated greater likelihood of biological activity [37,38]. Among the 1583 peptide segments matched in the database, 190 had PR values ≥ 0.50, with 21 having PR values ≥ 0.80. The basic characteristics of the PR ≥ 0.80 peptides are summarized in Figure 1E.

### 3.2. Scorpion Peptides Accelerate Wound Healing in Diabetic Mice by Suppressing Excessive Inflammation

**Figure 2:**
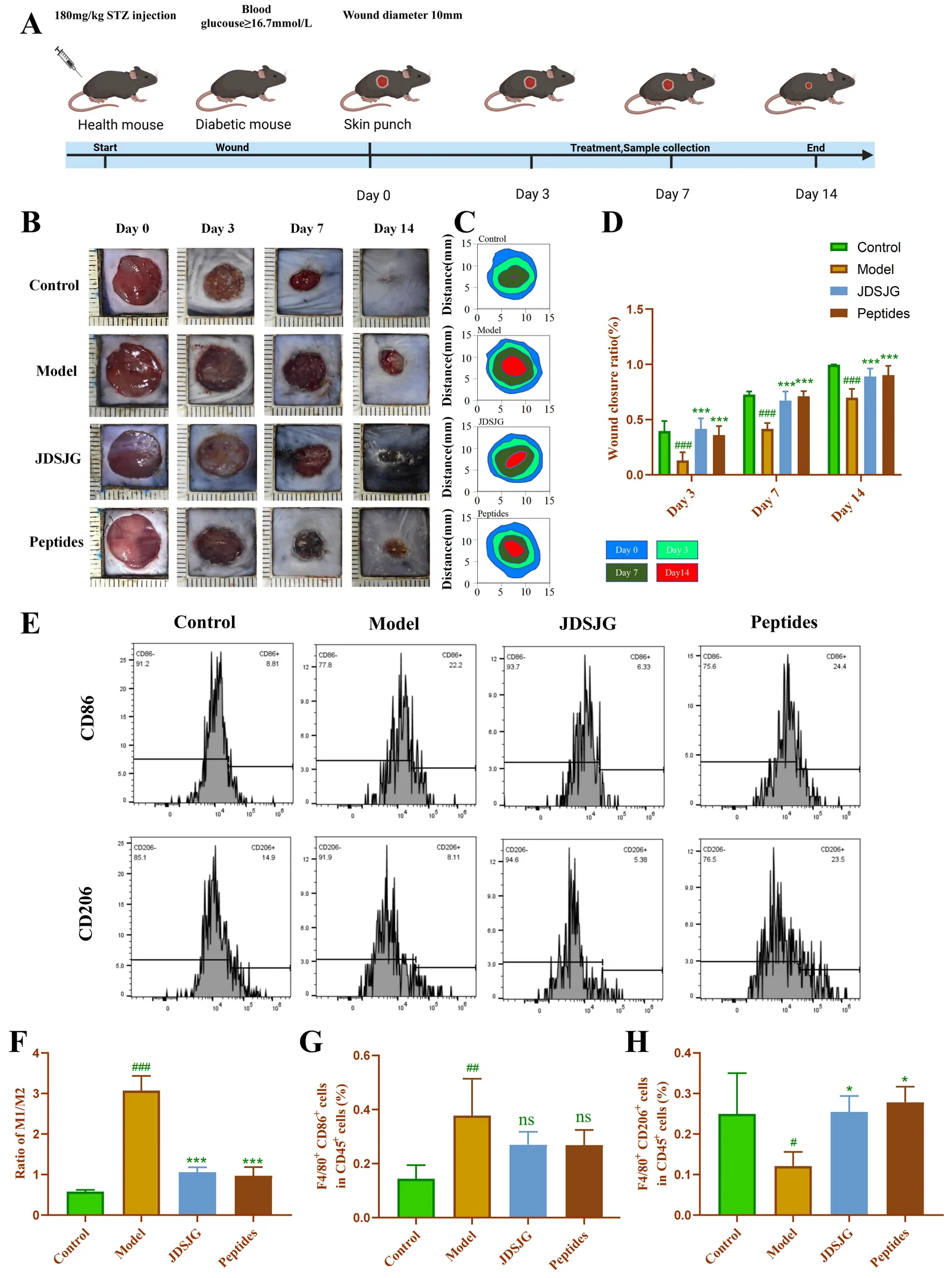
Scorpion peptides promotes wound healing in diabetic mice and regulates macrophage polarization. (**A**) Schematic of experimental procedures and sample collection. (**B**) Representative images of wound healing in mice treated with blank hydrogel, JDSJG, or scorpion peptides hydrogel at days 0, 3, 7, and 14. (**C**) Heatmaps of wound area at different time points. (**D**) Quantitative data of relative wound area at different time points (*n* = 6). (**E**) Flow cytometry analysis to detect M1 and M2 phenotype expression in macrophages within wound tissues across different groups (*n* = 3). (**F**) Quantification of M1/M2 expression. (**G**) Quantification of CD86 expression. (**H**) Quantification of CD206 expression. All data are presented as mean ± standard deviation, # *p* < 0.05, ## *p* < 0.01, ### *p* < 0.001 Normal group versus Model group, * *p* < 0.05, *** *p* < 0.001 Model group versus Drug group.

Diabetic wounds are closely associated with persistent inflammatory responses. To evaluate the efficacy of scorpion peptides on wound healing, this study established a full-thickness skin wound model in diabetic mice (Figure 2A). Mice were divided into control, model, JDSJG, and scorpion peptide groups. Wound healing was recorded at days 0, 3, 7, and 14 post-treatment. As shown in Figure 2B–D, wound healing progressed differently across groups with prolonged treatment. Both JDSJG and scorpion peptides significantly promoted wound healing in diabetic mice. Macroscopic examination of wounds at 14 days post-treatment revealed that wounds in the JDSJG and scorpion peptide groups were dry and scabbed over, with no significant perilesional swelling and well-epithelialized surfaces, indicating near-complete healing. In contrast, the model group exhibited minor exudate, mild perilesional swelling, and poor epithelialization, demonstrating markedly inferior repair compared to other groups (Figure 2B,C). Wound area quantification using ImageJ software revealed that at 3, 7, and 14 days post-treatment, the wound healing rate in the model group was significantly lower than that in the JDSJG and scorpion peptide groups. Furthermore, at 14 days post-treatment, the scorpion peptide group achieved a wound healing rate of 90.2%, the JDSJG group reached 89.1%, while the model group remained at only 69.7% (Figure 2D).

Delayed inflammatory responses and dysregulation of inflammatory mediators are key factors contributing to the poor healing of diabetic wounds. Macrophages, as crucial immune cells in the body, play vital roles in clearing infectious agents and mediating inflammatory responses. Based on their functional characteristics and surface markers, they are classified into classically activated M1 macrophages or alternatively activated M2 macrophages. Classically activated M1 macrophages are marked by specific proteins such as CD86 and iNOS, exhibiting pro-inflammatory properties that mediate ROS-induced tissue damage and impair tissue regeneration. Alternatively activated anti-inflammatory M2 macrophages are characterized by specific proteins like CD206 and CD163, possessing anti-inflammatory properties that promote wound healing by secreting anti-inflammatory factors and Vascular Endothelial Growth Factor (VEGF) to facilitate angiogenesis and collagen deposition. Therefore, we employed flow cytometry to detect macrophage phenotypes in wound tissues. Flow cytometry results demonstrated that both JDSJG and scorpion peptides significantly downregulated the M1/M2 ratio of macrophages in diabetic mouse wounds (Figure 2E,F). By inhibiting M1 macrophage polarization and promoting M2 polarization, these peptides effectively enhanced wound healing. Scorpion peptides significantly downregulated the M1/M2 ratio of wound macrophages in diabetic mice (Figure 2E,F). This effect was mediated by inhibiting M1 polarization and promoting M2 polarization, including downregulation of M1-associated markers (CD86) and upregulation of M2-associated markers (CD206) (Figure 2G,H). In summary, these findings suggest that scorpion peptides may alleviate inflammatory responses during skin wound healing by regulating macrophage polarization, thereby accelerating wound healing in diabetic mice.

### 3.3. Effects of Scorpion Peptides on the Phosphorylation Levels of the NF-κB Signaling Pathway and on Inflammatory Apoptosis

**Figure 3:**
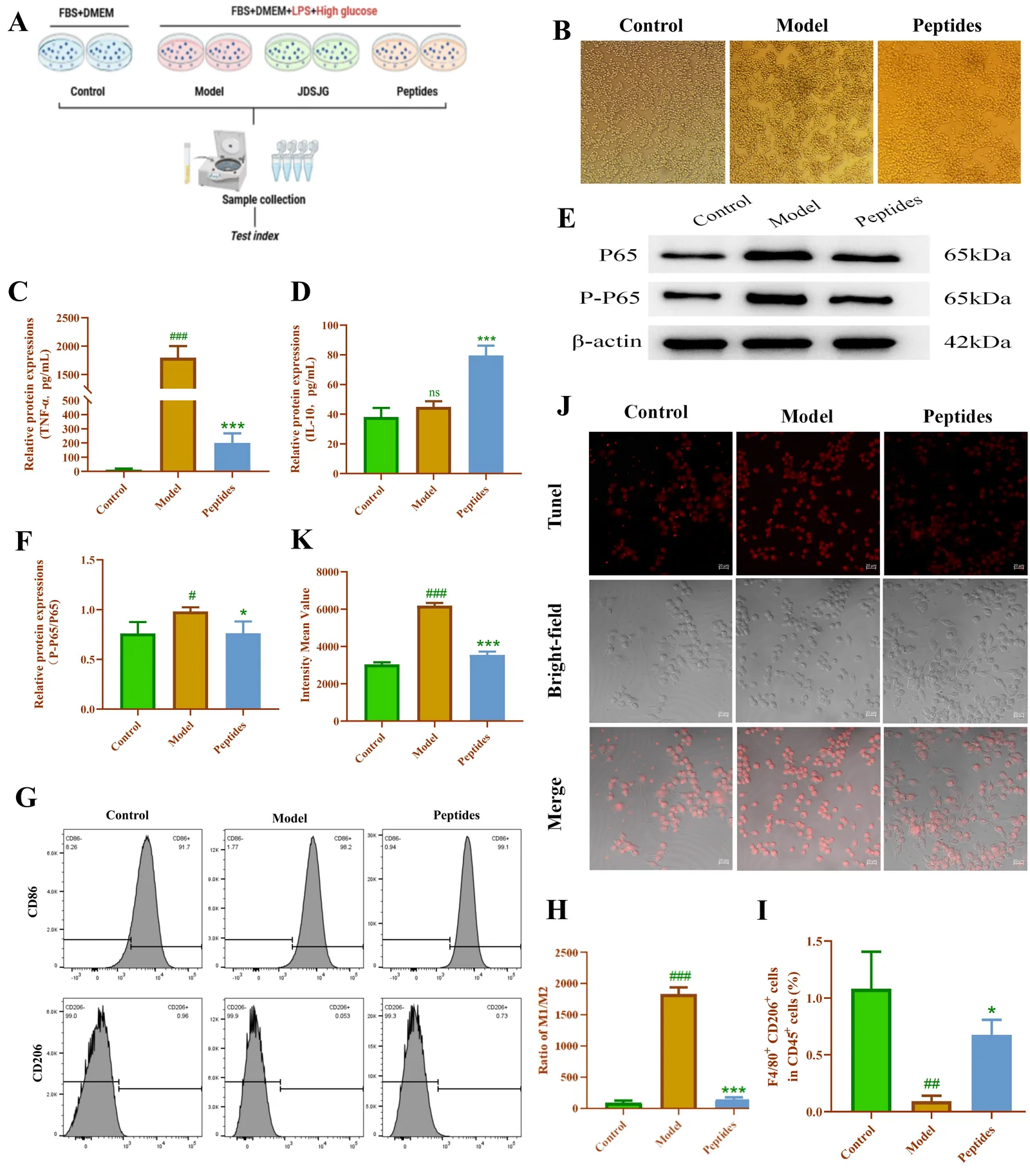
(**A**) Design of in vitro experiments to investigate how scorpion peptides regulate NF-κB phosphorylation, inflammatory factors, macrophage polarization, and apoptosis levels. (**B**) Microscopic morphology of macrophages. (**C**) Expression levels of inflammatory factor TNF-α in each group (*n* = 6). (**D**) Expression levels of inflammatory factor IL-10 in each group (*n* = 4). (**E**) WB analysis of P-P65/P65 protein expression in each group (*n* = 3). (**F**) Quantification of P-P65/P65 expression. (**G**) Flow cytometry analysis for detecting M1 and M2 phenotype expression in macrophages (*n* = 3). (**H**) Quantification of M1/M2 expression. (**I**) Quantification of CD206 expression. (**J**) TUNEL staining and scoring in each group (magnification = 20 μm, *n* = 3). (**K**) Quantification of mean TUNEL staining intensity. All data are presented as mean ± standard deviation, # *p* < 0.05, ## *p* < 0.01, ### *p* < 0.001 Normal group versus Model group, * *p* < 0.05, *** *p* < 0.001 Model group versus Drug group.

To further investigate the specific role of scorpion peptides in regulating macrophage polarization and promoting diabetic wound healing, we conducted additional in vitro experiments using RAW264.7 cells. These macrophages were either untreated or subjected to modeling intervention by adding LPS and high glucose to the culture medium, resulting in three groups: control, model, and scorpion peptides. Based on the CCK8 assay, the scorpion peptides intervention concentration was set at 2.5 μg/mL. Samples were collected from each group for subsequent analyses (Figure 3A). Macroscopic morphological changes in macrophages were observed under a microscope. Macrophages in the normal group exhibited an oval shape, while those in the model group displayed dendritic extensions. Compared to the model group, the scorpion peptide group showed reduced dendritic extension (Figure 3B). Supernatants were collected and analyzed by ELISA to measure inflammatory cytokine levels (TNF-α and IL-10) in macrophages from each group. Compared to the normal group, the model group showed significantly increased TNF-α levels with no statistically significant difference in IL-10 levels. Compared to the model group, the scorpion peptide group exhibited significantly decreased TNF-α levels and markedly increased IL-10 levels (Figure 3C,D). The NF-κB signaling pathway is widely recognized as a key regulator of transcription for multiple proinflammatory mediators and cytokines. In the classic NF-κB pathway, after upstream signals activate the IKK complex, its catalytic subunit, IKKβ, becomes phosphorylated, which in turn phosphorylates IκB. Phosphorylated IkB (PIkB) is degraded via the ubiquitin-proteasome pathway, releasing the NF-κB dimer. Subsequently, the P65 subunit becomes phosphorylated (P-P65) and translocates into the nucleus, initiating the transcription of pro-inflammatory genes. This leads to the excessive release of pro-inflammatory factors such as TNF-α and IL-6, causing macrophages to remain in the pro-inflammatory M1 phenotype for an extended period and preventing their conversion to the anti-inflammatory M2 phenotype, ultimately resulting in abnormal cytotoxicity and inflammatory responses.

Therefore, we investigated the effects of combined LPS and high-glucose intervention on NF-κB phosphorylation 24 h after treatment of macrophages. WB analysis was performed to detect the expression levels of P-P65/P65 proteins in each group (Figure 3E). Compared with the control group, the model group showed upregulated P-P65/P65 expression, whereas P-P65/P65 expression was suppressed following scorpion peptide intervention (Figure 3F). Flow cytometry analysis of macrophage phenotypic shifts (Figure 3G). Flow cytometry results indicate that scorpion peptides significantly downregulate the M1/M2 ratio in macrophages (Figure 3H) and promote M2 polarization, upregulating the expression levels of M2-associated markers (CD206) (Figure 3I). TUNEL staining further confirmed that scorpion peptides reversed the apoptotic effects of combined LPS and high glucose on macrophages (Figure 3J,K). These results suggest that the wound-healing effects of scorpion peptides may be related to their ability to improve the local inflammatory microenvironment; their mechanism of action is associated with regulating cytokine levels, promoting M2 polarization of macrophages, inhibiting NF-κB phosphorylation, and reducing macrophage apoptosis.

### 3.4. Peptide Activity Fragment Research of Scorpion Peptides Based on Peptidomics

**Figure 4:**
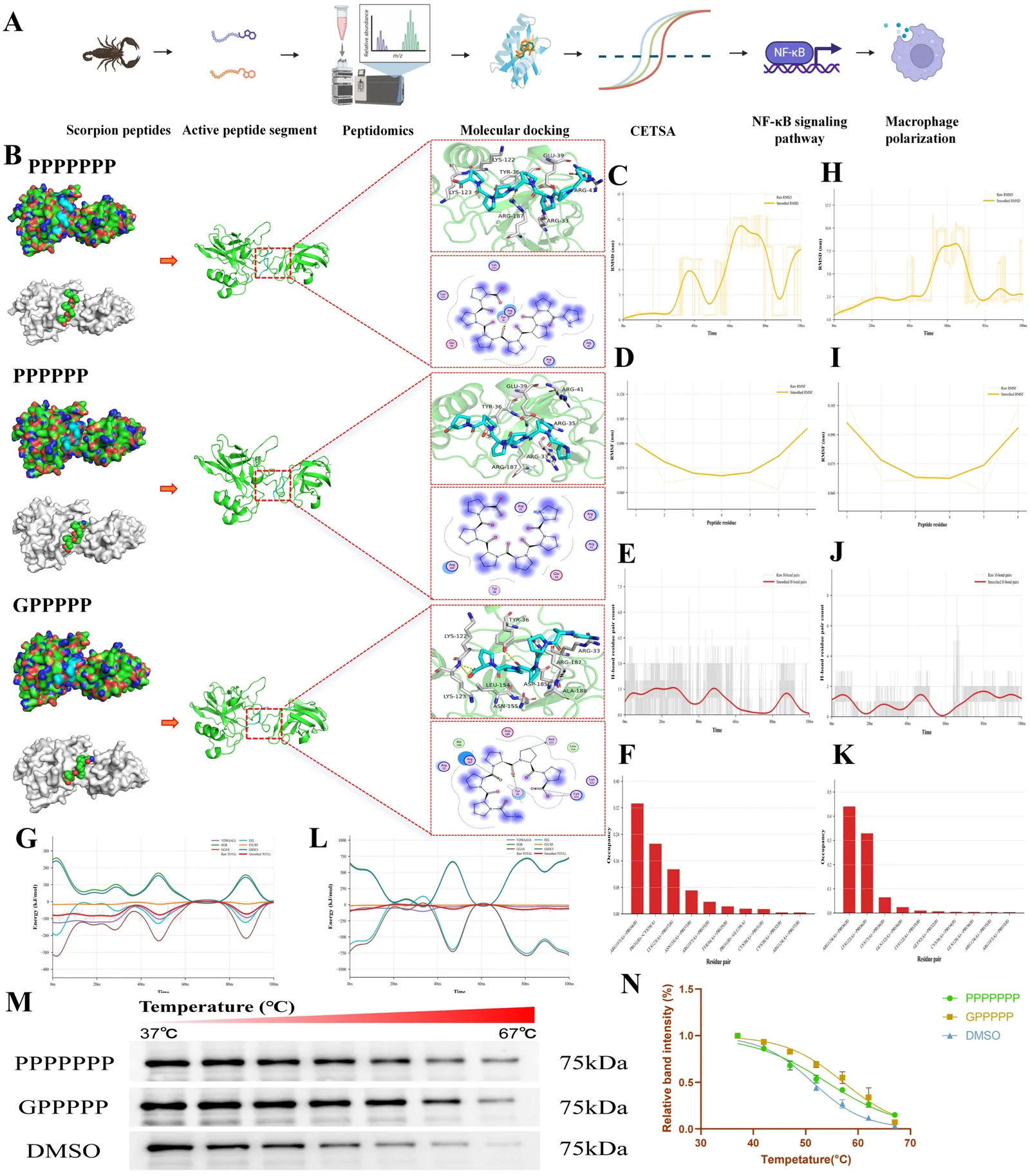
(**A**) Overall workflow diagram for scorpion peptides active site studies based on peptidomics. (**B**) Molecular docking results for P65 with PPPPPPP, PPPPPP, and GPPPPP. Left panel shows AlphaFold3-predicted binding conformations of PPPPPPP, PPPPPP, GPPPPP, and P65. Top-left shows molecular docking surface model of the active peptide segment and P65. Bottom-left shows molecular docking site map of the active peptide segment and P65. Center: Molecular ribbon diagrams of PPPPPPP, PPPPPP, GPPPPP, and P65. Blue ribbons represent the active peptide; green ribbons represent the RHD domain. The upper right and lower right corners present enlarged 3D and 2D views of the overall binding patterns for PPPPPPP, PPPPPP, GPPPPP, and P65. Light green indicates the P65 RHD domain, gray short rods represent interacting amino acids, blue short rods denote the active peptide segment, and yellow dashed short rods symbolize hydrogen bonds and salt bridges. (**C**–**G**) Trajectories of RMSD, RMSF, hydrogen bonding, and MM/GBSA binding free energy of PPPPPPP over time, and Top 10 H-bond Residue Pair Occupancy. (**H**–**L**) Trajectories of RMSD, RMSF, hydrogen bonding, and MM/GBSA binding free energy of GPPPPP over time, and Top 10 H-bond Residue Pair Occupancy. (**M**) Results of CETSA experiments with P-P65 and DMSO, PPPPPPP, and GPPPPP at six different temperatures (37, 42, 47, 52, 57, 62, 67 °C) (*n* = 3). (**N**) Melting curves of the P-P65 protein after treatment with DMSO, PPPPPPP, and GPPPPP.

Select the top 10 peptides with the highest activity for molecular docking with target protein P65. After preparing the ligand and receptor, configure the grid and docking settings to perform peptide-based molecular docking. Lower docking binding energy values indicate stronger interaction and higher compatibility between the target protein and the compound. The binding energy results revealed that the three peptides named PPPPPPP, PPPPPP, and GPPPPP exhibited the lowest binding energies, demonstrating favorable interactions and good compatibility with the core target protein (all binding energies below −5 kcal/mol) (Supplementary Table S1). Therefore, this study selected the PPPPPPP, PPPPPP, and GPPPPP peptides for molecular docking with the target protein P65 to investigate potential interaction mechanisms. The full-length sequences of the three short peptides and P65 were read separately, and their complex models were constructed using AlphaFold3. Each peptide yielded five predicted conformations. AlphaFold3 assigned a ranking score (0–1, higher values indicating superior quality) based on structural capability, prediction reliability, and conformational conflicts (Supplementary Table S2). Examining the binding patterns across 15 conformations revealed that all peptides interacted within the P65 19-306 region—specifically on the RHD domain—and exhibited highly consistent conformational behavior. Consequently, the model 0 conformation of each peptide was selected for binding mode analysis. PPPPPPP binds to the hinge region of the P65 RHD domain, which is predominantly composed of polar amino acids. The short peptide is driven by van der Waals interactions and forms a hydrogen bond with ARG187, LYS122, and LYS123, forming a hydrogen bond with ARG187. The PPPPPP sequence binds to the hinge region of the P65 RHD domain. Driven by van der Waals forces, the short peptide is surrounded by amino acids ARG41, ARG33, ARG35, GLU39, TYR36, and ARG187. The six-proline peptide does not form any hydrogen bonds. GPPPPP forms abundant hydrogen bonds with P65, involving three amino acids (ARG187, TYR36, and LYS123) that form three hydrogen bonds and one salt bridge with the short peptide. GPPPPP exhibits high binding affinity (Figure 4B).

Subsequently, molecular dynamics simulations were conducted to further evaluate the stability of the P65 protein complexes with the two peptide segments, PPPPPPP and GPPPPP (a summary of the PPPPPPP and GPPPPP systems is provided in Supplementary Table S3). Inactive peptides, which had been previously confirmed as such through experiments or experimental design, were defined as negative controls (see Supplementary Figure S3 for the Protein RMSD and RMSF results for PPPPPPP and GPPPPP). The binding of PPPPPPP to P65 exhibited relatively limited fluctuations. The late-stage mean RMSD for the peptide was 6.335, the late-stage mean hydrogen bond value was 0.571 MM/GBSA, and the late-stage mean RMSF was −34.293, with a high occupancy rate for ARG187(A) → PRO4(B) (Figure 4C–G). The binding of GPPPPP to P65 is relatively stable, with minimal fluctuations in protein conformation. It exhibits a low and stable protein/peptide RMSD, a more consistent number of hydrogen bonds, two high-frequency interface anchors (ARG124(A) → PRO6(B) and LYS122(A) → PRO6(B)), and a consistently negative MM/GBSA energy over the final 25 ns (Figure 4H–L). This indicates that the P65-GPPPPP system retains distinct interface anchors and a relatively favorable energy state even after undergoing conformational adjustments. The H-bond occupancy results for PPPPPPP and GPPPPP are shown in Supplementary Table S4.

To evaluate the affinity between the P-P65 protein and the active peptide segment, we performed a CETSA assay (Figure 4M). The melting temperatures (Tm) for each group were calculated by fitting the normalized band intensities to a four-parameter logistic model (with the lower and upper bounds constrained to 0 and 1, respectively). Compared with the DMSO control group, treatment with both PPPPPPP and GPPPPP caused a rightward shift in the melting curves of the P-P65 protein, indicating enhanced thermal stability. The Tm value of the P-P65 protein increased from 51.35 °C in the DMSO control group to 54.04 °C in the PPPPPPP group and 57.21 °C in the GPPPPP group, respectively. These CETSA results confirm that PPPPPPP and GPPPPP bind directly to the P-P65 protein, with GPPPPP exhibiting a stronger stabilizing effect (Figure 4N).

We selected PPPPPPP and GPPPPP two peptides for subsequent validation. High-performance liquid chromatography (HPLC) analysis confirmed that both PPPPPPP and GPPPPP peptide extracts had a purity exceeding 95% and were soluble in ultrapure water.

### 3.5. Effects of Active Peptide Fragments on the Phosphorylation Levels of the NF-κB Signaling Pathway and on Inflammatory Apoptosis

**Figure 5:**
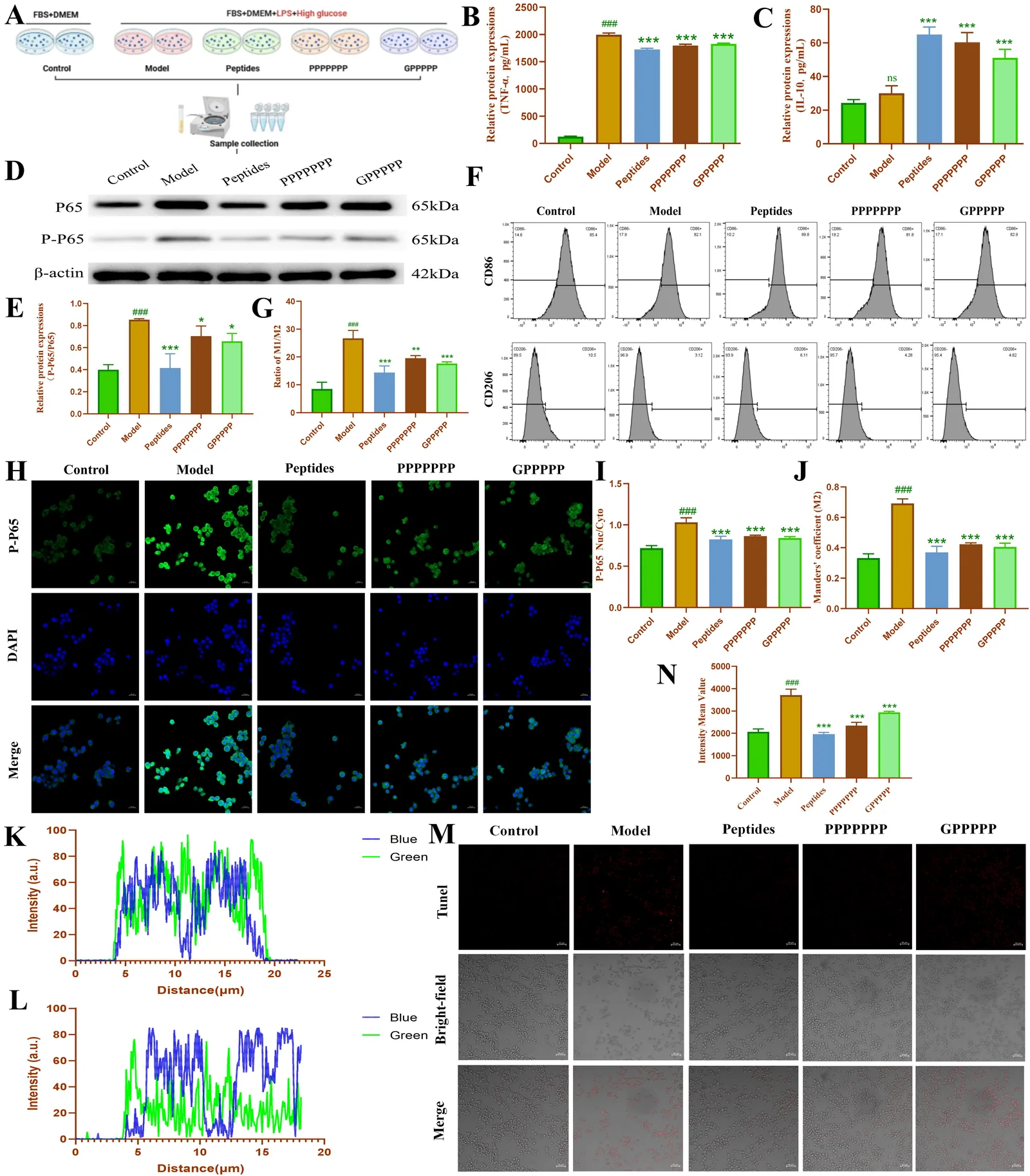
(**A**) Experimental design to test whether active peptide fragments regulate M1/M2 polarization of macrophages via the NF-κB pathway, alleviate inflammatory responses, and reduce macrophage apoptosis. (**B**) Expression levels of inflammatory factor TNF-α in each group (*n* = 3). (**C**) Expression levels of inflammatory factor IL-10 in each group (*n* = 4). (**D**) WB analysis of P65 and P-P65 protein expression in each group (*n* = 3). (**E**) Quantification of P-P65/P65 expression. (**F**) Flow cytometry analysis for detecting M1 and M2 phenotype expression in macrophages (*n* = 3). (**G**) Quantitative analysis of M1/M2 expression. (**H**) Cells were fixed, permeabilized, and stained for NF-κB P-P65 (green) and DAPI (blue) (scale bar = 20 μm, *n* = 3). (**I**) Statistics for the P-P65 nuclear-to-cytoplasmic ratio. (**J**) MCC analysis of the immunofluorescence co-staining for P-P65 and the cell nucleus (*n* = 3). (**K**) Colocalization analysis of P-P65 and DAPI in the model group. (**L**) Colocalization analysis of P-P65 and DAPI in the treatment group. (**M**) TUNEL staining and scoring in each group (scale bar = 50 μm, *n* = 3). (**N**) Quantitative analysis of mean TUNEL staining intensity. All data are presented as mean ± standard deviation, ### *p* < 0.001 Normal group versus Model group, * *p* < 0.05, ** *p* < 0.01, *** *p* < 0.001 Model group versus Drug group.

To further investigate the specific roles of the PPPPPPP and GPPPPP peptide segments in regulating macrophage polarization and promoting diabetic wound healing, we conducted subsequent in vitro experiments using RAW264.7 cells. We cultured RAW264.7 cells and divided them into control, model, scorpion peptides, PPPPPPP, and GPPPPP groups. Samples were collected from each group for relevant assays (Figure 5A). The CCK8 assay measured macrophage survival rates after exposure to different concentrations of active peptide fragments. Results showed PPPPPPP achieved the highest survival rate of 140% at 5 μg/mL, while GPPPPP reached 120% at the same concentration (Supplementary Figure S4). Microscopic examination revealed morphological changes in macrophages across groups. Normal group macrophages exhibited an oval shape, while model group macrophages displayed dendritic extensions. Compared to the model group, both PPPPPPP and GPPPPP groups showed reduced numbers of dendritic extensions (Supplementary Figure S5). The supernatants were collected and the levels of proinflammatory factors TNF-α and IL-10 produced by macrophages in each group were detected by ELISA. Compared with the normal group, the model group showed significantly increased TNF-α levels and no statistically significant difference in IL-10 levels. Compared with the model group, the scorpion peptides, PPPPPPP, and GPPPPP groups exhibited significantly reduced TNF-α levels and significantly increased IL-10 levels (Figure 5B,C). WB analysis detected P-P65/P65 protein expression levels in each group (Figure 5D). Compared with the control group, the model group exhibited upregulated P-P65/P65 expression. Compared with the model group, P-P65/P65 expression was suppressed following intervention with scorpion peptide, PPPPPPP, and GPPPPP (Figure 5E). Flow cytometry was used to assess macrophage phenotypic shifts (Figure 5F). Flow cytometry results showed that intervention with scorpion peptide, PPPPPPP, and GPPPPP significantly downregulated the M1/M2 ratio in macrophages (Figure 5G). Immunofluorescence was used to examine P-P65 nuclear translocation (Figure 5H). Compared with the control group, both the P-P65 nucleus-to-cytoplasm ratio and the M2 colocalization coefficient were significantly elevated in the model group. This M2 coefficient, calculated based on the Manders’ Colocalization Coefficient (MCC), reflects the extent of P-P65 coverage within the cell nucleus. (Figure 5I,J). In the colocalization scatter plots, the green (P-P65) and blue (DAPI) peaks showed a high degree of overlap (Figure 5K). Following intervention with scorpion peptide, PPPPPPP, or GPPPPP, both the P-P65 nuclear-cytoplasmic ratio and the M2 coefficient decreased significantly (Figure 5I,J), and the peaks in the colocalization scatter plots showed a tendency to separate (Figure 5L). TUNEL staining further confirmed that scorpion peptides, PPPPPPP, and GPPPPP reversed the apoptotic effects of combined LPS and high glucose on macrophages (Figure 5M,N).

These results indicate that the two active peptide segments, PPPPPPP and GPPPPP, can inhibit the phosphorylation of the NF-κB P65 subunit, Inhibition of P-P65 Nuclear Translocation Induced by LPS and High Glucose, effectively promote the conversion of macrophages from the M1 to the M2 phenotype, alleviate excessive inflammatory responses, and exert an anti-apoptotic effect. Scorpion peptides showed no significant difference from PPPPPPP and GPPPPP in suppressing the inflammatory factor TNF-α, indicating that the monomers possess fundamental anti-inflammatory activity. However, scorpion peptides outperformed the monomers in promoting IL-10 expression, reducing the P-P65/P65 ratio, exerting anti-apoptotic effects, and regulating M1/M2 polarization. This demonstrates that scorpion peptides, as a complex, possesses superior anti-inflammatory and signaling pathway modulation capabilities, offering more comprehensive immune regulation and cellular protection.

## 4. Discussion

Diabetic wounds exhibit delayed healing, potentially leading to skin infections and even a series of complications such as localized necrosis and amputation. This significantly impacts patients’ quality of life and may even threaten their health, making it a major clinical concern [39,40]. A moderate inflammatory response plays a multifaceted positive role in wound repair [41]. It not only aids in clearing necrotic tissue and preventing infection but also promotes tissue repair and regeneration by secreting growth factors and regulating cellular functions [42,43]. However, in diabetic conditions, the transition from pro-inflammatory to anti-inflammatory states during wound repair is impaired [44]. Tissues remain in a chronic inflammatory state, hindering the initiation of repair processes and obstructing normal tissue regeneration and repair, resulting in persistent non-healing wounds [45]. Therefore, reducing excessive inflammatory responses at the wound site is a core component of diabetic wound repair. Inflammatory responses permeate the entire diabetic wound healing process. In the early inflammatory phase, damaged cells release signals to recruit neutrophils into the wound for pathogen elimination [7,46]. Circulating monocytes migrate to the wound site, differentiate into macrophages, and undergo polarization to perform neutrophil-like phagocytic functions. Macrophages constitute a vital component of the innate immune system, playing a crucial role in combating microbial infections [47]. Two distinct macrophage phenotypes exert pro-inflammatory and anti-inflammatory functions, respectively, through the synthesis and release of various chemical mediators [48].

JDSJG demonstrates excellent therapeutic effects on diabetic skin wounds, with scorpion serving as the effective anti-inflammatory component within JDSJG. Scorpion peptides, as the primary medicinal active constituents of scorpions, exhibit significant anti-inflammatory, antimicrobial, and immunomodulatory functions. Therefore, we hypothesize that scorpion peptides represent bioactive peptides with therapeutic potential. As one of Earth’s oldest organisms, scorpions comprise a diverse group of over 2000 species across 16 families [49,50]. Within this vast scorpion population, species like Hottentotta zagrosensis and the Mexican scorpion are unsuitable for direct medicinal use or long-term herbal applications due to their extreme toxicity and high risk [51,52,53]. Only a limited number of species are considered medically significant. The scorpion raw materials used in this experiment originated from Luoyang, China. The Pharmacopoeia of the People’s Republic of China stipulates that the sole botanical source for scorpion is the East Asian scorpion Buthus martensii Karsch (BmK) from the family Buthidae. BmK possesses high concentrations of active components, moderate and controllable toxicity, high safety, and significant medicinal value. In China, large-scale artificial breeding techniques for BmK are well-established, enabling stable supply of quality-controlled raw materials with distinct industrial advantages, making it an optimal source species for scorpion medicinal materials [54,55]. Peptides derived from BmK have been reported to exhibit broad pharmacological activities. Current research on BmK primarily focuses on its roles as a voltage-gated ion channel modulator, analgesic, and antitumor agent [21,55]. In oncology, BmK peptides AGAP, AK, and GK have been demonstrated to inhibit inflammatory responses, cell proliferation, and apoptosis, thereby exerting antitumor effects [56,57]. In neuroscience, BmKNT1, BmKNT2, Dfsin 4, and SVHRSP exert analgesic and anticonvulsant effects by regulating voltage-gated calcium, sodium, and potassium channels [58,59,60]. This study selected BmK as the research subject, expanding the potential of scorpion peptides for treating inflammatory wounds and opening new avenues for developing novel anti-inflammatory drugs.

This study employed ultrafiltration to extract peptide components from BmK. De novo sequencing based on LC-MS/MS, combined with database searches (Peaks 8 software), enabled systematic identification of peptide sequences, followed by screening for active peptide segments. Based on molecular docking results, the active peptides PPPPPPP and GPPPPP were synthesized for subsequent functional validation. In a constructed full-thickness skin defect model of diabetic mice, scorpion peptides demonstrated significant wound-healing activity. Mechanistic studies have shown that the anti-inflammatory effects of scorpion peptides and their active peptide fragments are closely related to the regulation of macrophage polarization and are accompanied by a significant downregulation of phosphorylation levels in the NF-κB pathway, suggesting that this pathway may be involved in the remodeling of the wound immune microenvironment. ELISA results confirmed that scorpion peptides and its active peptide segments effectively modulate inflammatory factor expression in RAW264.7 cells stimulated with LPS and high glucose in vitro. They suppress pro-inflammatory cytokine TNF-α expression while promoting anti-inflammatory cytokine IL-10 expression. Flow cytometry results demonstrated that scorpion peptides and their active peptide segments significantly increased CD206 expression, inducing macrophage polarization toward the M2 type. TUNEL staining confirmed that scorpion peptides and their active peptide segments effectively reversed macrophage apoptosis under high-glucose conditions, exerting a cytoprotective effect. This study confirms that scorpion peptides effectively reverse the imbalanced polarization state of macrophages in diabetic wounds through anti-inflammatory and pro-repair mechanisms. However, this study focused on the regulatory effects of scorpion-derived peptides on inflammatory responses and the molecular mechanisms underlying the NF-κB pathway, confirming that these peptides can effectively inhibit the expression of pro-inflammatory factors and reduce local inflammatory cell infiltration at both in vivo and in vitro levels. However, the direct effects of these peptides on repair processes—such as tissue re-epithelialization, collagen deposition and remodeling, and angiogenesis—during the later stages of wound healing have not been systematically evaluated within the scope of this study and require further investigation. In summary, the anti-inflammatory, M2-polarizing, and anti-apoptotic effects of scorpion peptides are closely related to their inhibition of NF-κB signaling pathway activation, suggesting that the NF-κB signaling axis may be one of the key molecular targets through which scorpion peptides exert their multifaceted protective effects.

## 5. Conclusions

Through peptidomics, animal models, and cellular experiments, this study conducted a preliminary exploration of scorpion peptides and their active components. Their wound-healing effects are associated with improvements in the local inflammatory microenvironment, and the inhibition of the NF-κB signaling pathway may be involved in this process. The study highlights the potential of scorpion peptides in the development of drugs for treating inflammatory wounds and provides preliminary experimental evidence for developing scorpion peptides as a novel immunomodulatory therapeutic strategy for diabetic wounds.

## Data Availability

The datasets supporting the conclusions of this article are included within the article and its additional files.

## Supplementary Materials

The following supporting information can be downloaded at: https://www.mdpi.com/article/10.3390/biomedicines14092071/s1, Figure S1: Changes in body weight and blood glucose levels (*n* = 6). (A) Changes in body weight. (B) Changes in blood glucose levels. All data are presented as mean ± standard deviation, ### *p* < 0.001, Normal group versus Building Module group. Figure S2: Specific gating strategies and representative flow cytometry plots. (A) Wound tissue flow cytometry flow circle gate representative diagram. (B) Cell flow cytometry flow circle gate representative diagram. Table S1: Molecular docking binding energy results. Table S2: Conformation ranking scores for PPPPPPP, PPPPPP, and GPPPPP. Table S3: Summary of the PPPPPPP and GPPPPP systems. (A) PPPPPPP H-bond occupancy. (B) GPPPPP H-bond occupancy. Figure S3: The Protein RMSD and RMSF results. (A) RMSD and RMSF results for PPPPPPP protein. (B) RMSD and RMSF results for GPPPPP protein. Table S4: The H-bond occupancy results. (A) PPPPPPP system summary. (B) GPPPPP system summary. Figure S4: CCK8 assay measured macrophage survival rates. (A) PPPPPPP CCK8 Result Diagram. (B) GPPPPP CCK8 Result Diagram. Figure S5: Microscopic morphology of macrophages.

## Abbreviations

BMK	Buthus martensii Karsch
DUs	Diabetic ulcers
JDSJG	Jie Du Sheng Ji Gao
LPS	Lipopolysaccharide
IL-10	Interleukin-10
NF-κB	Nuclear factor kappa-B
STZ	Streptozotocin
TNF-α	Tumor necrosis factor-α
WB	Western Blot
