# Supplementary material for: Mechanism and Component Study of Scorpion Peptides Regulation of Macrophage Polarization in Diabetic Wounds
Source: Biomedicines. 2026 Sep 15;14(9):2071. doi: 10.3390/biomedicines14092071 (PMC13604153; doi:10.3390/biomedicines14092071)
Supplement: Supplementary file 1 [file biomedicines-14-02071-s001.zip › biomedicines-4428304-supplementary.pdf]

(A)

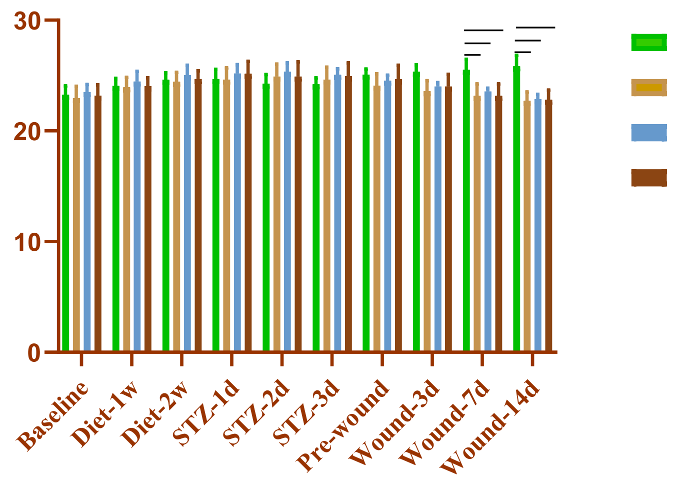

(B)

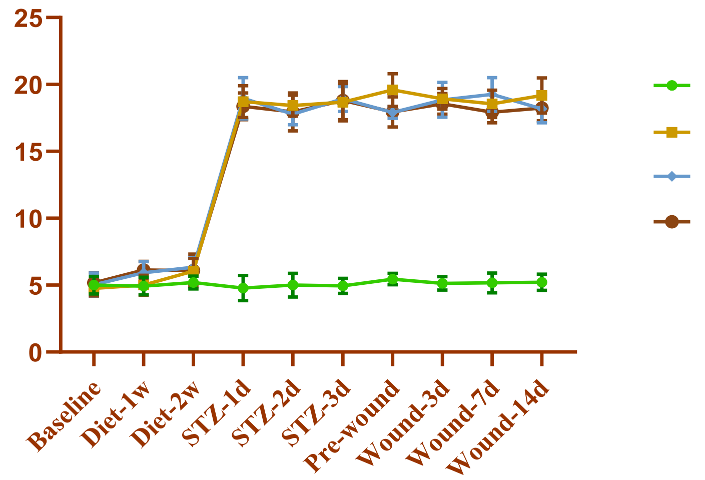

Figure S1

Changes in body weight and blood glucose levels (*n*=6). **(A)** Changes in body weight. **(B)** Changes in blood glucose levels.All data are presented as mean ± standard deviation, ### $p < 0.001$, Normal group versus Building Module group.

(A)

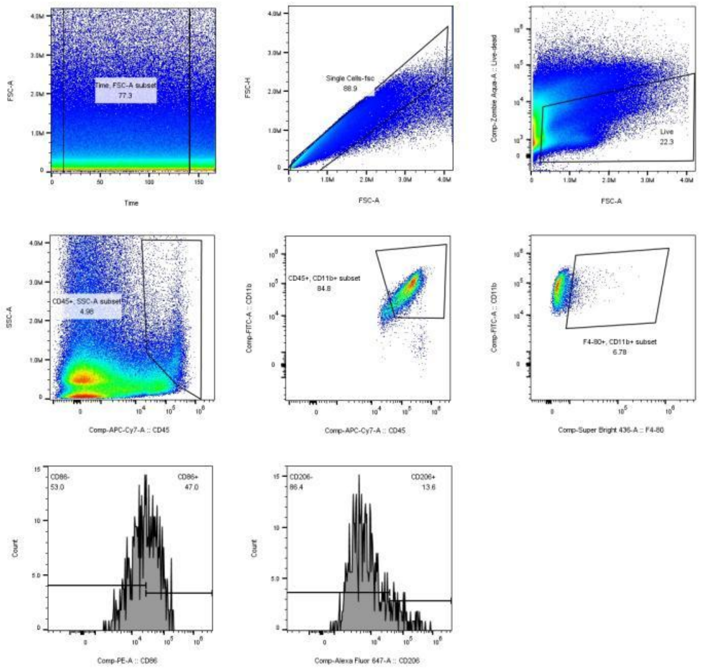

(B)

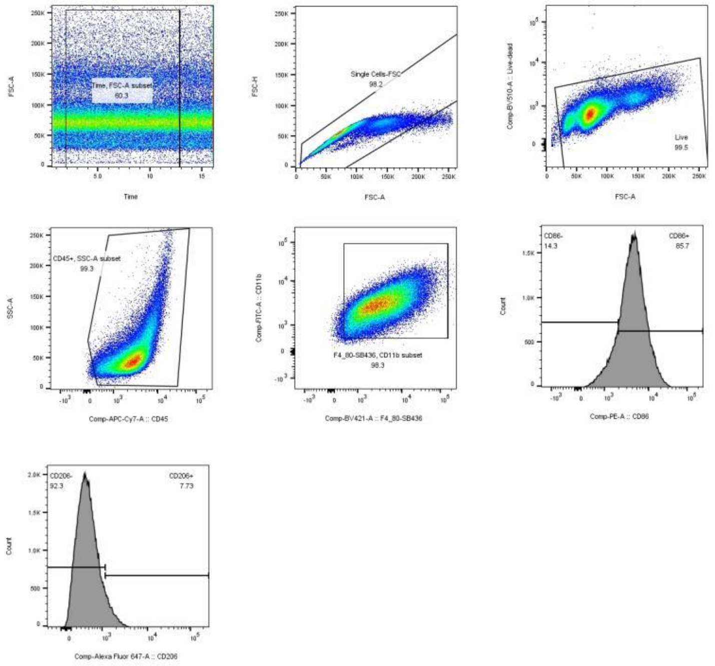

Figure S2

Specific gating strategies and representative flow cytometry plots. **(A)** Wound tissue flow cytometry flow circle gate representative diagram. **(B)** Cell flow cytometry flow circle gate representative diagram.

Peptide	P65（Unit:kcal/mol）
PPPPPPP	−10.04
PPPPPP	9.46
GPPPPP	−7.73
GFGGFH	−0.73
APGPINF	−0.87
FSYGYNF	+2.13
GPFPEPL	−0.35
PTPYLF	−4.46
FGHLFK	+0.25
GFGKFH	+1.19

Table S1 Molecular docking binding energy results.

Model \ Peptide	PPPPPPP	PPPPPP	GPPPPP
Model 0	0.79	0.78	0.81
Model 1	0.79	0.78	0.80
Model 2	0.77	0.78	0.79
Model 3	0.75	0.77	0.76
Model 4	0.73	0.72	0.74

Table S2 Conformation ranking scores for PPPPPPP, PPPPPP, and GPPPPP.

(A)

Ranking	Residue pair	Occupancy rate(%)
1	ARG124(A)−>PRO6(B)	33.30
2	PRO1(B)−>CYS38(A)	21.16
3	LYS123(A)−>PRO7(B)	13.45
4	ASN155(A)−>PRO7(B)	7.07
5	ARG187(A)−>PRO5(B)	3.64

(B)

Ranking	Residue pair	Occupancy rate(%)
1	ARG124(A)−>PRO6(B)	44.01
2	LYS122(A)−>PRO6(B)	32.90
3	LYS37(A)−>PRO6(B)	6.45
4	GLN132(A)−>PRO6(B)	2.42
5	LYS122(A)−>PRO5(B)80	0.99

Table S3

Summary of the PPPPPPP and GPPPPP systems. **(A)** PPPPPPP H-bond occupancy. **(B)** GPPPPP H-bond occupancy.

(A)

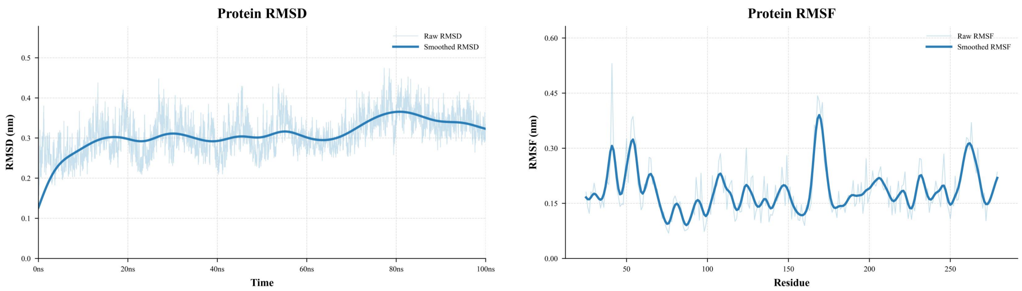

(B)

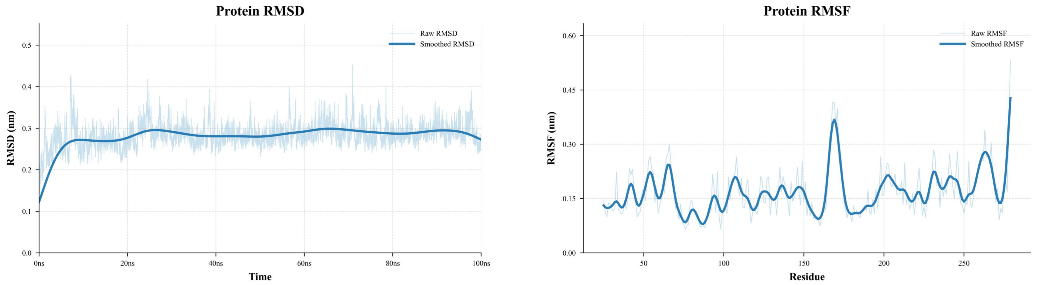

Figure S3

The Protein RMSD and RMSF results. **(A)** RMSD and RMSF results for PPPPPPP protein. **(B)** RMSD and RMSF results for GPPPPP protein.

(A)

Time interval	Mean value of Protein RMSD(nm)	Mean value of Peptide RMSD(nm)	Mean value of H-bond	Mean value of MM/GBSA TOTAL (kJ/mol)
0–25 ns	0.278	0.630	1.421	−79.968
25–50 ns	0.303	3.408	1.166	−56.491
50–75 ns	0.311	8.193	0.485	−17.672
75–100 ns	0.350	6.334	0.571	−34.293

(B)

Time interval	Mean value of Protein RMSD(nm)	Mean value of Peptide RMSD(nm)	Mean value of H-bond	Mean value of MM/GBSA TOTAL (kJ/mol)
0–25 ns	0.271	1.636	0.815	−34.806
25–50 ns	0.284	2.304	0.933	−31.048
50–75 ns	0.292	6.800	0.585	−19.733
75–100 ns	0.291	2.738	1.477	−65.016

Table S4

The H-bond occupancy results. **(A)** PPPPPPP system summary. **(B)** GPPPPP system summary.

(A)

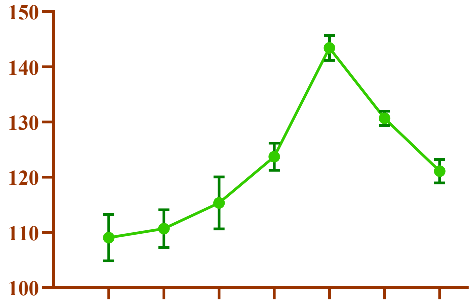

(B)

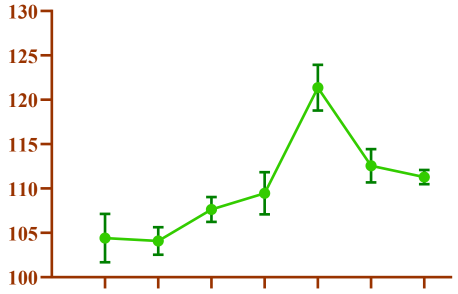

Figure S4

CCK8 assay measured macrophage survival rates. **(A)** PPPPPPP CCK8 Result Diagram. **(B)** GPPPPP CCK8 Result Diagram.

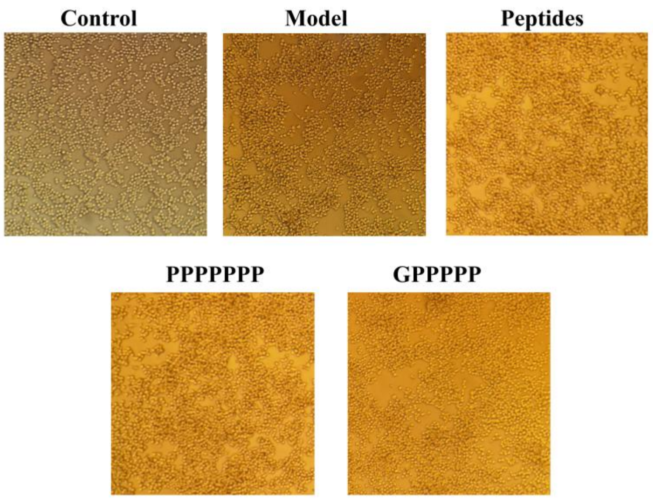

Figure S5 Microscopic morphology of macrophages.
